# Measuring Surface Pressure on Rotating Compressor Blades Using Pressure Sensitive Paint

**DOI:** 10.3390/s16030344

**Published:** 2016-03-08

**Authors:** Markus Pastuhoff, Nils Tillmark, P. Henrik Alfredsson

**Affiliations:** Competence Center for Gas Exchange (CCGEx), KTH Mechanics, SE-100 44 Stockholm, Sweden; markuspa@mech.kth.se (M.P.); nt@mech.kth.se (N.T.)

**Keywords:** pressure sensitive paint, PSP, turbo charger, compressor

## Abstract

Pressure sensitive paint (PSP) was used to measure pressure on the blades of a radial compressor with a 51 mm inlet diameter rotating at speeds up to 50 krpm using the so called lifetime method. A diode laser with a scanning-mirror system was used to illuminate the paint and the luminescent lifetime was registered using a photo multiplier. With the described technique the surface-pressure fields were acquired for eight points in the compressor map, useful for general understanding of the flow field and for CFD validation. The PSP was of so called fast type, which makes it possible to observe pressure variations with frequencies up to several kHz. Through frequency spectrum analysis we were able to detect the pulsating flow frequency when the compressor was driven to surge.

## 1. Introduction

To determine the pressure on a surface, the pressure sensitive paint (PSP) technique is an attractive possibility. PSP is an optical technique whereby the pressure distribution on a surface can be obtained without the use of mechanical pressure transducers. This technique has several advantages as compared to standard pressure measuring techniques, e.g., the spatial resolution can be high and it can be applied to models or structures where standard pressure taps are complicated or impossible to install, such as thin blades and/or rotating components.

In the present work we describe the use of PSP on the impeller blades of a small-scale compressor intended for use in the gas-exchange system of a passenger car. In the gas-exchange system the compressor is used to increase the pressure and thereby the density of the air (mixture) supplied to the cylinders of the engine. Usually the compressor is one part of a turbocharger, where a turbine driven by the engine exhaust flow is mounted on the same axis and provides the power to turn the compressor wheel. Other solutions also exist, for example, it can be driven mechanically by the engine shaft itself or by an electrical motor.

**Figure 1:**
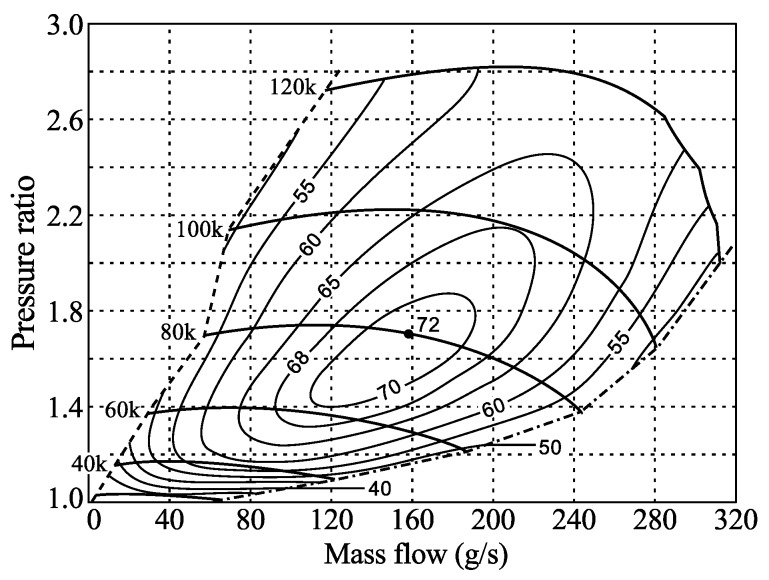
A typical compressor map for a passenger car.

The compressor map is usually used to describe its performance which gives the pressure ratio over the compressor as a function of the mass flow (see Figure 1) for various rotational speeds, marked in this map by their values in rpm ranging from 30 k to 120 k. In the map the efficiency lines, *i.e.*, efficiency as compared to ideal isentropic efficiency, are also shown as contour lines labeled from 40% to 72%. Rotational speeds for passenger cars may reach about 200 krpm, whereas compressors for heavy-duty trucks may go up to 100 krpm. The map is limited to the right in the diagram by the so called choke line and to the left by the surge line. At the choke line the Mach number reaches one and the flow is choked, whereas at the surge line the flow in the compressor becomes separated (often called rotating stall) and may result in a flow with low frequency variations. At surge the compressor ceases to function as such and continuous use in this regime may lead to compressor failure.

In the present work we are applying fast responding PSP in order to show that it is possible to obtain the pressure distribution on a compressor impeller at high rotational speeds and to detect the surge phenomenon. Section 1 gives an introduction to the PSP technique, whereas Section 2 describes the experimental setup as well as the preparation of the PSP and application of it on the impeller. Section 2.1 describes the methodology for the data acquisition and Section 2.2 the image data processing and conversion into the pressure distribution using the calibration (shown in Section 2.3). Finally the results are presented and discussed in Section 3 and the conclusions are given in Section 4.

### Pressure Sensitive Paint

Pressure sensitive paint is an optical technique for measuring air pressure on surfaces and is well described in the literature, for instance, in the monograph by Liu and Sullivan [1]. The sensing part of PSP consists of luminescent molecules that are either bound to a surface using an oxygen-permeable binder or directly deposited on the, usually porous, surface; the term “paint” is used regardless. The luminescent molecules, or luminophores, are excited to higher energy states by the absorption of colliding photons, and as the luminophores return to their base energy level, excess energy is released as photons with longer wavelengths, since some energy is lost as heat in the process. This wavelength separation, or Stokes shift, is an important property since it enables the separation of excitation and emission light through the use of optical filters.

What enables the luminophores to be used as pressure sensors is the mechanism of oxygen quenching; as oxygen molecules collide with the luminophores in their excited state the excess energy of the excited luminophores can be transferred to the oxygen in a non-radiative process. With an increase in the amount of surrounding oxygen, the probability of a single luminophore being quenched within a fixed period of time increases. As a result the luminescent intensity of a population of luminophores under continuous excitation light will decrease. Also, the average lifetime of a population of luminophores in their excited state will shorten when the probability of oxygen quenching increases.

In PSP, the luminophores are embedded in an oxygen-permeable binder where, according to Henry’s law, the concentration of oxygen is directly proportional to the partial pressure of oxygen in the surrounding gas. In this way pressure is measured through oxygen concentration under the requirement that the gas contains oxygen and also that the concentration of oxygen in the gas is constant throughout the experiment.

The luminescent intensity, *I*, and average lifetime, *τ*, of PSP decrease as pressure goes up. This relationship is described by the Stern-Volmer equation in the form

(1) $$\frac{I_{ref}}{I}=\frac{\tau_{ref}}{\tau }=A+B\frac{p}{p_{ref}}$$

where $I_{ref}$ and $\tau_{ref}$ are the reference intensity and average lifetime sampled at a reference pressure $p_{ref}$. The two coefficients, *A* and *B*, are temperature dependent and determined through calibration. In practice, when using Equation (1) as a calibration curve, a third term, $C{\left(p/p_{ref}\right)}^{2}$, is often added to the right hand side.

In PSP experiments, one has the option of using either the intensity method, measuring the intensity of the luminescent emission; or the lifetime method, measuring the luminescent lifetime of the emission. Using the intensity method, the paint layer is continuously illuminated, using light-emitting diodes (LEDs) or other stable short wavelength sources, and the luminescence is acquired using a digital imaging sensor. In contrast, when using the lifetime method the paint is usually excited using a short pulse of high intensity light and the average lifetime, or time constant, of the exponential decay of the luminescence is measured using either a photo multiplier for point measurements, or high-speed digital imaging sensors for full-field measurements. Since most luminophores exhibit lifetimes of the order of microseconds, full-field lifetime measurements put high demands on both the acquisition and illumination systems.

The intensity reference, $I_{ref}$, is needed since the luminescent intensity is susceptible to inhomogeneities in the paint layer and in the illumination. Furthermore, the paint intensity will decrease in time as individual luminophores break down due to heat and photo bleaching, even during the course of the experiment, making it important *when* the reference is acquired.

For the lifetime method a reference time constant, $\tau_{ref}$, is used. This is however less critical since the lifetime is independent of the peak intensity level, making measurements sensitive only to paint inhomogeneities.

**Figure 2:**
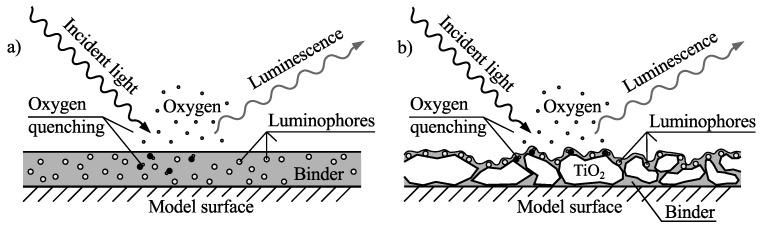
(**a**) Traditional and (**b**) Polymer/Ceramic pressure sensitive paint (PSP). Adopted from Reference [2].

Traditional PSP, shown in Figure 2a, consists of luminophores in a polymer binder and is usually applied to the surface of interest by spray painting. It can give accurate results since a high concentration of luminophores per surface area is possible, resulting in a high signal-to-noise ratio. The trade-off is slow response times, on the order of seconds, as oxygen needs time to penetrate the binder. Since this is a diffusion process the response time of the paint is proportional to the square of its thickness and in order to decrease the response time the paint can be made thinner, but at the expense of lower luminescent intensity.

In order to decrease response times while keeping the luminescent intensities at acceptable levels polymer/ceramic PSP (PC-PSP) has been developed. PC-PSP falls in the group of fast response time PSPs thoroughly described in References [3,4]. Here a base coat containing ceramic particles is applied to the surface, let to dry, and coated with a thin layer of luminophores in a solvent. Variants where the luminophores are mixed in with the ceramic coating exist as well. The thin layer contributes to a cut-off frequency on the order of several kHz, and through the surface roughness of the base coat a high concentration of luminophores is achieved. An illustration of PC-PSP is shown in Figure 2b.

Examples of the use of PSP in compressor research are sparse and have so far been restricted to the use of the intensity technique for acquiring data. Liu *et al.* [5] describes pressure measurements on a 0.3 m diameter axial compressor using a scanning laser system and a photomultiplier tube. Navarra *et al.* [6] measured pressure on a 0.7 m diameter axial compressor using a pulsed laser with an expanded beam and a CCD camera, whereas Gregory *et al.* [7] studied a radial compressor with a 50 mm inlet diameter using a pulsed LED array and a CCD camera.

## 2. Experimental Section

**Figure 3:**
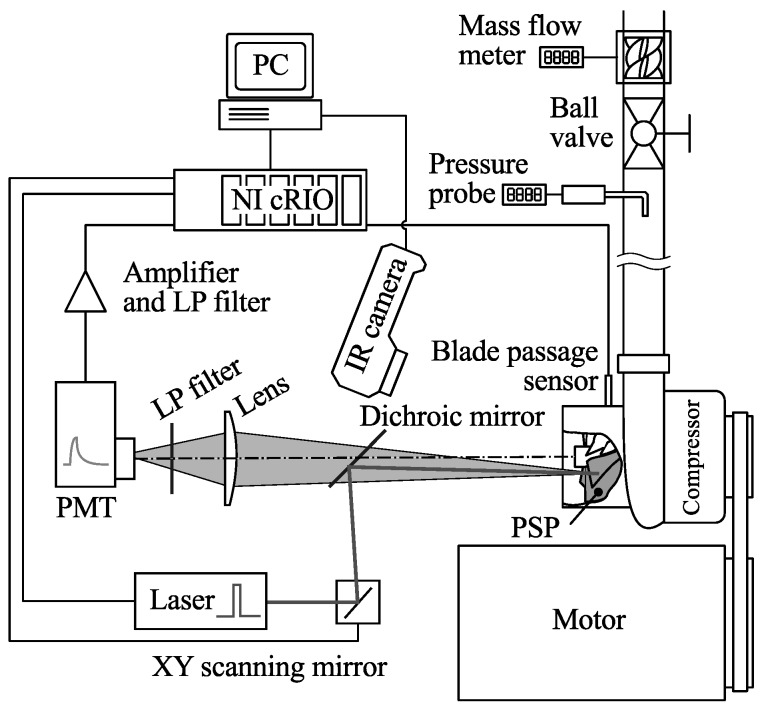
Scanning laser setup.

**Figure 4:**
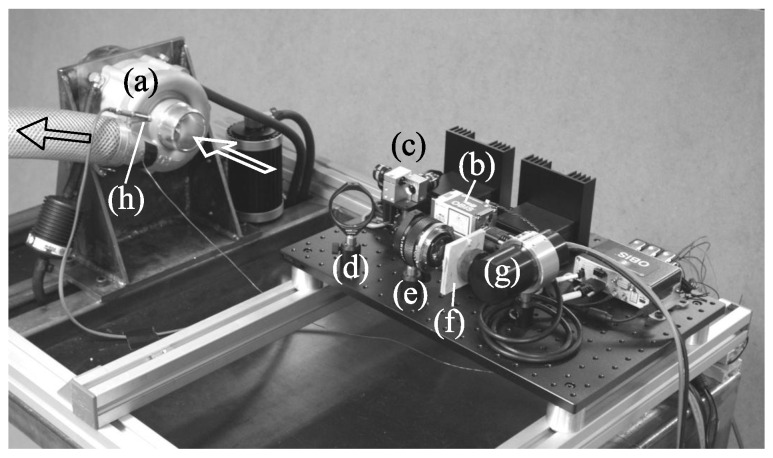
Photograph of the experimental setup with (**a**) compressor; (**b**) diode laser; (**c**) scanning galvanic mirrors; (**d**) dichroic mirror; (**e**) lens; (**f**) long-pass filter; (**g**) photo-multiplier tube; and (**h**) blade passage sensor.

The experiments were performed in the CCGEx Cicero lab located at KTH. An overview of the setup is shown in Figure 3 and a photograph in Figure 4. The compressor under investigation was a 51 mm inlet diameter Rotrex C30-74 with an aluminum impeller with seven primary and seven splitter blades. It was driven by a 45 kW electric motor via a belt with a drive ratio of 1:4. The compressor is further geared internally with a drive ratio of 1:9.49. The inlet of the compressor was open to the atmosphere and the outlet was connected to a plastic hose. About 30 hose diameters downstream the compressor outlet a section with a Pitot tube (to measure the stagnation pressure), a rotary ball valve to control the mass flow and a turbine mass flow meter (GL Flow Gx FL1) was installed. An optical blade passage sensor was mounted in the compressor housing (Optel-Thevon 152G10) to measure the impeller rotation rate and phase, whereas a 60×60 pixel thermographic camera (FLIR i3) was used in order to measure the impeller surface temperature.

The seven primary blades of the compressor impeller were instrumented with a polymer/ceramic pressure sensitive paint (PC-PSP) consisting of two layers, a ceramic undercoat and the sensor layer itself. The undercoat was prepared as described by Reference [3], by mixing water, titanium oxide, and dispersant (Rohm & Haas Duramax D-3005) at a weight ratio of 1:1.72:0.012. The mixture was ball milled for one hour before adding 3.5 wt % of polymer binder (Rohm & Haas Duramax B-1000).

The mixture was applied to the compressor blades using a spray gun (Aztek A220) in thin cross-coating layers until good coverage was achieved. After applying the undercoat and letting it dry for 10 min, the sensor layer, consisting of platinum meso-tetra(pentafluorophenyl)porphyrin (PtTFPP) solved in methanol (0.2 mg/mL), was applied in a similar fashion. For the purpose of paint calibration a small aluminum plate was painted in parallel.

In order to collect the emitted light from the PSP a photo-multiplier tube was placed centered on the rotational axis of the compressor behind a 590 nm long-pass filter (Schott OG590) and a focusing lens. Excitation light was provided by a 405 nm diode laser (Coherent OBIS 405LX). While not strictly semantically being a pulsed laser, it has the ability to be digitally modulated up to 100 MHz. The laser light was lead into the same optical path as the acquisition system by a long-pass (505 nm cutoff) dichroic mirror (Thorlabs DMLP505L) mounted between the compressor and the focusing lens. The laser spot was guided to a point on the impeller surface through two scanning galvanic mirrors (Thorlabs GVSM002/M).

Control of the laser pulse and the galvanic mirrors, as well as acquisition of the photo-multiplier tube and the blade passage sensor was provided via a National Instruments (NI) CompactRIO system consisting of a chassis with a built in real-time computer and programmable logic (NI cRIO 9068), a digital I/O module (NI 9401, 0.1 μs update rate), an analog input module (NI 9201, 12 bit, 800 kS/s), and an analog output module (NI 9263, 16 bit, 100 kS/s).

The output signal from the photomultiplier was fed through an inverting amplifier (based on operational amplifier INA111 configured with an inverting gain of 300, and a cutoff frequency of about 200 kHz) before sampling.

### 2.1. Data Acquisition

**Figure 5:**
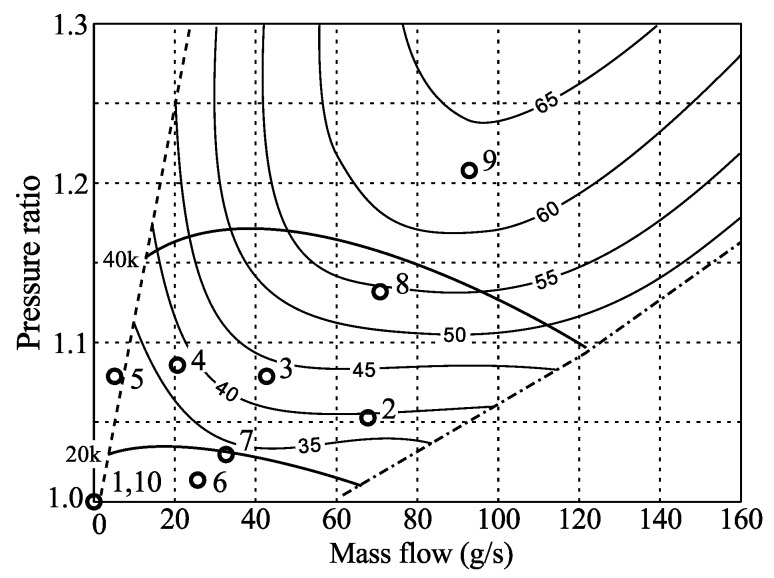
Rotrex C30-74 compressor map as provided by manufacturer with the tested points shown as (${}^{\circ }$).

Ten cases (numbered 1–10) were measured and are tabulated in Table 1, where the relevant flow parameters are given. The different cases are also shown together with the manufacturer supplied compressor map in Figure 5 for easy reference. The numbering is in chronological order and all measurement shown here were taken during a time of about one hour. Although deviations from the speed lines can be seen (best seen for case 8), some discrepancy between the manufacturer supplied and measured parameters are expected since the compressor inlet conditions are different.

The first case, sampled without flow, was used to calculate a reference time constant of the luminescent decay, $\tau_{ref}$ and the last case, also without flow, was used as a control to validate the calibration. For cases 2–5 the rotational speed was held constant while the mass flow was decreased in steps until the compressor was well into surge (case 5). For cases 6–9 the intention was to increase the rotational speed in steps while staying inside the compressor map until some part of the system could no longer keep up. In the end the limiting factor was the optical blade-passage sensor that got coated by a fine white dust from the ceramic PSP undercoat.

The acquisition of each flow case followed the same routine where first the rotational speed of the impeller was set by adjusting the speed of the electric motor while monitoring the signal output of the blade-passage sensor through a frequency counter. The mass flow was set by adjusting the valve located downstream the compressor while the mass-flow meter display was observed, and values for mass flow and downstream stagnation pressure were recorded before starting the PSP acquisitions.

**Figure 6:**
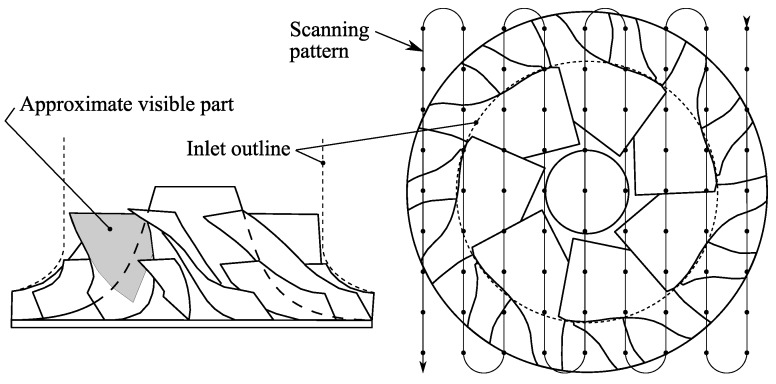
Overview of compressor wheel illustrating visible area and scanning pattern, with reduced number of points for visibility.

The laser-scanning system was programmed to scan the surface of the impeller in 401×401 points. Starting from the top right, seen from the laser side, the points were scanned alternately down and up while moving to the left as illustrated in Figure 6. For each point the scanning mirrors guided the laser spot to the corresponding coordinate on the compressor impeller and the system was set to trigger on a blade passage. (In the non-rotational cases the system was simply set to continue without trigger.) On triggering, a single 1 μs laser pulse was shot right after the start of the sampling of the photomultiplier signal such that the first point acquired was taken before the laser pulse. This initial sample was used to remove any background light in the signal. For each of the 401×401 spatial points, 30 temporal points were acquired at a sampling frequency of 800 kHz. The time to scan the impeller varies from about 30 s at 50 krpm to two minutes in the 10 krpm case. In this way the whole temporal behavior of the PSP was recorded and extraction of time constants were left to post-processing.

**Figure 7:**
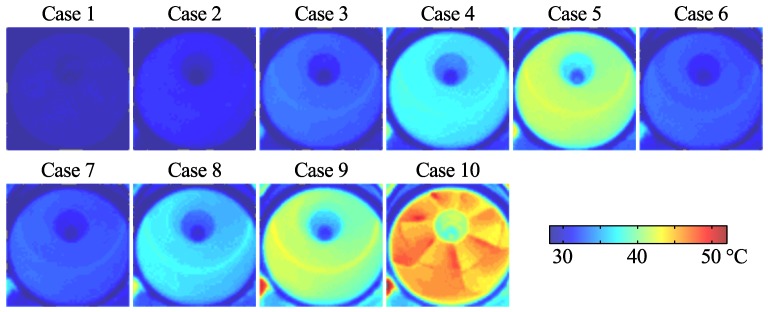
Thermal images of impeller at indicated flow cases.

Immediately after the PSP sampling the impeller was brought to rest and a thermal image of the compressor was taken in order to obtain the impeller surface temperature. Thermal images of the 10 cases are shown in Figure 7.

Since temporal information and blade identity were lost using the scanning measurements described above, frequency information cannot be obtained. To obtain frequency information single spot measurements can be used and we demonstrate this for two flow cases, namely cases 3 and 5 referring to Table 1. Case 3 was at normal operation close to maximum efficiency inside the compressor map, and case 5 at surge condition. The data acquisition followed the same procedure as the scanning laser measurements with the difference that the laser spot was kept stationary and measurements were taken at the center of the visible part, as indicated in Figure 6, on each of the impeller blades resulting in seven samples per impeller revolution.

### 2.2. Post Processing

For each scanning laser experiment, the acquired data set contained the temporal behaviors for each of the 401×401×30 acquired points. The data set was reorganized into 30 images where each image resembled an image of the whole impeller at one point in time. It was noted that the scaling of the scanning mirror was slightly different between the *x* and *y* axes and each image was squeezed using bicubic interpolation to 401×385 pixels to fit the geometry of the compressor wheel. Finally each image was cropped around the compressor wheel resulting in images of 343×343 pixels. In the final conversion to pressure only the first, fourth, and fifth of theses images were used, as will be further explained in this section.

For the stationary laser experiments, where acquisition was at a single point in space, no image re-scaling was necessary and the data sets were simply treated as series of 160,801 samples measured on each blade in sequence.

**Figure 8:**
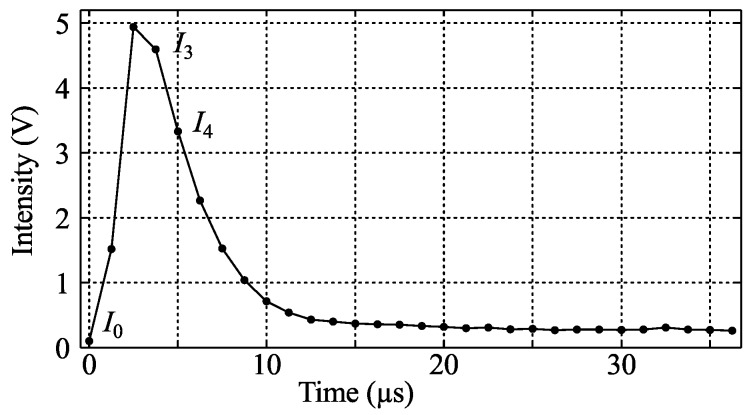
Typical temporal behavior of luminescent decay.

An example of the temporal behavior of a single pixel is shown in Figure 8. The luminescent intensity of the PSP is expected to decay exponentially, and if the time axis is shifted such that t=0 just after the laser pulse has ended, this can be formulated as

(2) $$I\left(t\right)=I\left(0\right)\;e^{-t/\tau }$$

where *τ* is the time constant, or mean lifetime, of the decay. A standard approach to evaluate *τ* would be to use a least squares fit to several points of the sampled data in order to reduce uncertainty. However, as a satisfactory high signal-to-noise ratio was already reached, a two-point method was used instead in order to limit the rotational blur during the evaluation time to a maximum of 0.4${}^{\circ }$, at 50 krpm. In these experiments *τ* was calculated from points $I_{3}$ and $I_{4}$, as shown in Figure 8, by first subtracting background noise, $I_{0}$, and putting the values into Equation (2) together with $t_{3}$ and $t_{4}$. Elimination of I(0) and solving for *τ* gives:

(3) $$\tau =\Delta t/\ln\left(\frac{I_{3}-I_{0}}{I_{4}-I_{0}}\right)$$

where $\Delta t=t_{N+1}-t_{N}$.

Having reduced the data to two-dimensional matrices of size 343×343 containing values of *τ*, pressures were calculated from

(4) $$\frac{\tau_{ref}}{\tau }=A\left(T\right)+B\left(T\right)\frac{p}{p_{ref}}+C\left(T\right){\left(\frac{p}{p_{ref}}\right)}^{2}$$

where $p_{ref}$ and $\tau_{ref}$ are reference pressures and time constants from the first flow case where the impeller was stationary. Here, $p_{ref}$ is the atmospheric pressure and $\tau_{ref}$, being pixel specific, was obtained by image rotation of the *τ*-matrix from case 1 (using a nearest neighbor algorithm) to spatially match the *τ*-matrix of the evaluated case. For case 1 itself, the result would simply be a matrix of ones, so instead it was divided by itself with a $0.1^{\circ }$ rotation in order to observe the effects of small rotational errors, which turned out to be about $0.02\;p_{atm}$ with a 95% confidence interval, taken from the standard deviation of the surface pressure.

The three calibration coefficients *A*, *B*, and *C* are temperature dependent and the thermal images (Figure 7) were used to determine temperature on the blade surface. It was assumed that the blade temperatures were homogeneous and the median value of the parts of the thermal images covering the blade was used as temperature. Corresponding values are shown in Table 1.

Values for *A*, *B*, and *C* were determined from a paint sample in a separate calibration chamber as described in the following section.

### 2.3. PSP Calibration

**Figure 9:**
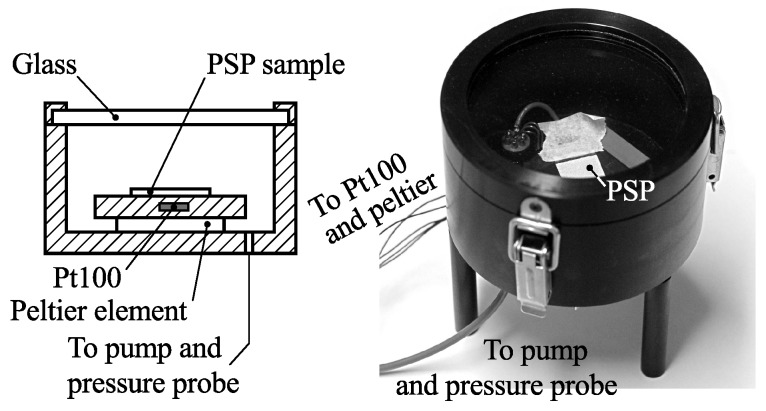
Calibration chamber.

Calibration was done separately from the experiments on a paint sample using a calibration chamber (Figure 9) where pressure and temperature can be controlled independently. The paint was excited, and emission was sampled using the same setup as the main experiment (Figure 3) by simply placing the chamber in front of the compressor inlet and scanning the surface of the paint sample. Pressure was varied between 90 and 120 kPa in steps of 5 kPa, and temperature was varied between 20 and 40 ${}^{\circ }C$ in steps of 5 ${}^{\circ }C$ as measured by the internal temperature probe of the calibration chamber.

As mentioned earlier the temperature of the impeller was determined by an IR camera which needed to be calibrated since the emission factor of the PSP was unknown. Therefore the paint sample in the chamber was measured by the same thermal imaging camera at several known temperatures and could thereby be used as a reference for the impeller temperature. This was done at atmospheric pressure and without the front glass of the chamber since it is not transparent to IR light.

**Figure 10:**
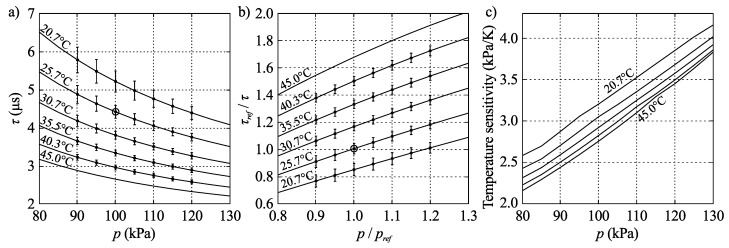
Calibration data. (**a**) Measured points with 95% confidence intervals; (**b**) Best fit to Equation (4) with reference point circled; (**c**) Temperature sensitivity.

For each calibration point, time constants for 70×70 points on the PSP surface were computed according to Equation (3), and from the obtained time constants mean values and 95% confidence intervals were calculated. These are plotted in Figure 10. In the same figure calibration data are also plotted according to Equation (4) as well as as the temperature sensitivity of the PSP.

## 3. Results and Discussion

**Figure 11:**
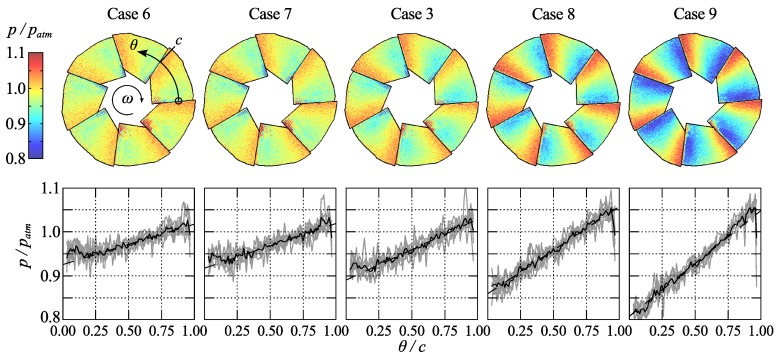
Pressure distribution on the impeller blades (upper row of figure) from scanning measurements for cases 6, 7, 3, 8 and 9, *i.e.*, with increasing rotational speed. The lower row of the figure shows the pressure along the chord, defined in case 6 as c=2π/7, of the blade at half the blade radius, grey lines show the distribution from the seven individual blades, black line their average.

Surface-pressure maps acquired using the scanning-laser measurements are shown in Figure 11 for the five cases along the maximum efficiency line, defined as the line connecting the points of maximum efficiency for each (constant) speed line. Furthermore, the pressure along the chord of each individual blade at half the radius (single pixel values shown as grey lines in the right hand side figures) along with the mean of the seven distributions (black line) are also shown. As can be clearly observed the pressure increases almost linearly along the blades and the gradient increases as the rotational speed increases.

**Figure 12:**
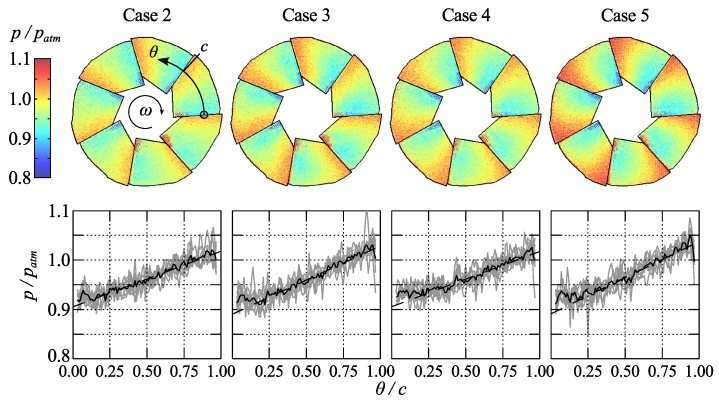
Pressure distribution on the impeller blades (upper row of figure) from scanning measurements for cases 2, 3, 4 and 5, *i.e.*, with decreasing flow rate at a constant rotational speed. The lower row of the figure shows the pressure along the chord, defined in case 2 as c=2π/7, of the blade at half the blade radius, grey lines show the distribution from the seven individual blades, black line their average.

A similar pressure plot is shown in Figure 12 for the case when the compressor is run at a constant speed but the flow rate is decreased until the surge line is reached.

In both figures small paint defects on the innermost part of the leading edges on three of the blades appears as spots of higher or lower pressure on all blades due to the used data acquisition method. Furthermore, a sharp drop in pressure can be observed at the trailing edge of each blade, where it is overlapped by the leading edge of the following blade. This is caused by spatial blur due to the width of the laser spot.

The difficulty in accurately determining the blade temperatures is considered to be the main source of systematic error in the presented data. Here the median value of the measured temperature on the blade surfaces obtained from the IR measurements shown in Figure 7 were used to correct the PSP data. The same IR images can also be used in an attempt to determine the uncertainty of the data by observing the variation of the temperature within the impeller part of the images, which, for the rotating cases, varies from ±0.25 ${}^{\circ }$C in case 2 to ±1 ${}^{\circ }$C in case 9. The latter corresponds to a pressure error of about 3.5 kPa, referring to the calibration data in Figure 10. Admittedly, it can be questioned whether this is an accurate method considering the internal reflections in the impeller. An additional source of systematic error stems from the width of the laser spot, which measures approximately 1 mm in diameter. This corresponds to an angular width of about 5.7${}^{\circ }$ at the inner part of the impeller and about 2.3${}^{\circ }$ at the outer part. This error is however only significant at the edges between two adjacent blades.

The stochastic part of the errors in these experiments is mainly comprised of read-out noise from the photomultiplier and the determination of *τ*. This noise is illustrated with 95% confidence intervals in Figure 10 and is limited to about 3.5 kPa over the experimental range. This estimation compares well with the variation of pressure shown along the chords in Figure 11 and Figure 12. An additional source of error is the uncertainty in angular position, which can be roughly estimated to 1${}^{\circ }$ from the edge sharpness between two blades. However, this has a small effect in terms of pressure due to the smooth variation of pressure over the blades.

**Figure 13:**
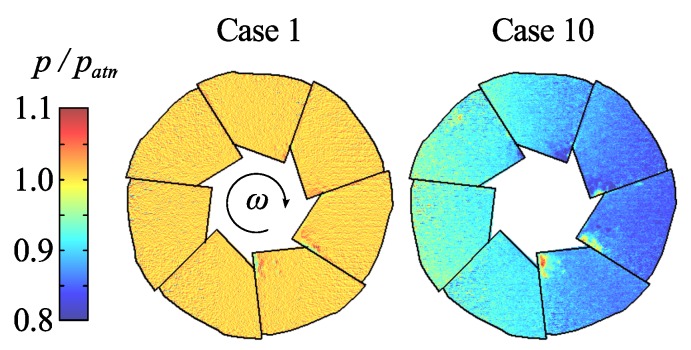
Pressure from scanning measurements for cases 1 and 10, *i.e.*, with the impeller at rest.

In order to further estimate the temperature effect, the pressure on the blades was measured both before and after the test series with the impeller at rest and thereby at atmospheric pressure. The results are shown in Figure 13. As expected before the run the impeller shows a uniform pressure as measured by the PSP. However, as the rotating impeller, cooled by the surrounding air flow, is stopped, it is heated from the inside. When the blades are scanned (from top-bottom-top-*etc.* and from right to left in Figure 13), the temperature is rising due to the heating from the gear box, which reached temperatures around 70 ${}^{\circ }$C during operation. Thus the impeller would have a higher temperature at the end of the scan (*i.e.*, left side in Figure 13). This would manifest itself as an apparent increase in pressure and explains the global pressure gradient seen over the impeller. An explanation to the low average pressure of case 10 can be given by the fact that the impeller temperature is not registered until after the PSP surface has been scanned, when the impeller has been even further heated, thus the blade temperature during pressure acquisition is lower than what is measured, which in turn would manifest as an apparent decrease in pressure. It is further observed from case 10 that the apparent pressure distribution on each individual blade is rather constant, supporting our previous assumption of a homogeneous blade temperature.

**Figure 14:**
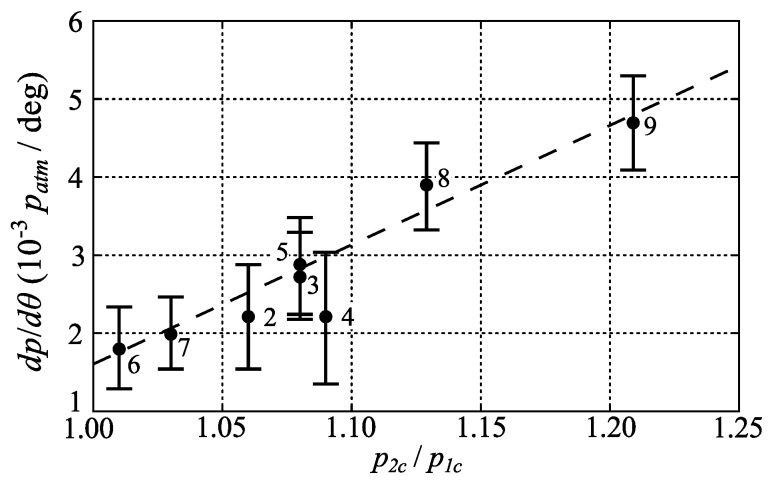
Pressure gradient on the center portion of the compressor blades (0.25<θ/c<0.75) as function of the measured pressure ratio over the compressor for cases 2–9. Same data as in Figure 11 and Figure 12.

In Figure 14 the pressure gradient along the central part of the chord is plotted against the pressure ratio across the compressor. For the cases close to the maximum efficiency line (*i.e.*, cases 6, 7, 3, 8, 9) there seems to be a linear variation of the pressure gradient with the pressure ratio which indicates that the pressure measurements in general are correct and specifically that the temperature correction procedure is sound. The error bars indicates the 95% confidence interval based on the gradient measurements from the seven different blades. For the measurements along the constant speed line (2, 3, 4, 5) the trends are not as obvious, however close to surge one would expect the flow to be less predictable.

**Figure 15:**
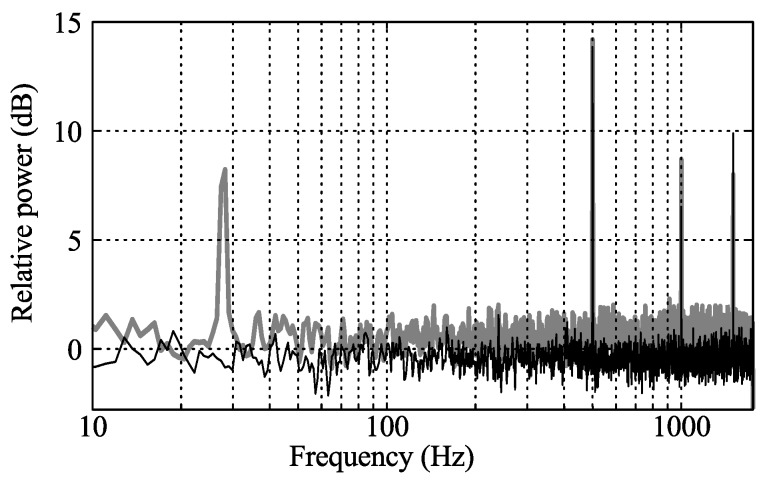
Power spectra of laser point measurements (arbitrary ordinate scale). Case 3 (*black*) and case 5 (*gray*).

From the stationary laser point measurements the power spectrum is plotted in Figure 15 for cases 3 and 5. In both cases peaks are seen at 500, 1000, and 1500 Hz. These peaks are believed to originate from small differences in the PSP coating between the individual impeller blades, where 500 Hz is the rotational frequency and the higher frequencies are harmonics. For case 3, measured inside the compressor map, the spectra is otherwise flat. For case 5 a clear surge frequency is seen at 28 Hz.

In this work a fast-type PC-PSP has been used for all measurement cases. While this approach has been necessary in order to detect the surge frequency, it could be discussed whether a traditional type PSP could have been used for the stable stationary cases. However, as a result of using a fast PSP we can conclude that the pressure distribution is independent of the blade position inside the compressor and not significantly affected by e.g., the diffuser tongue. Using a slow PSP with a response time in the order of seconds the pressure shown would be the average over a revolution and any such spatial inhomogeneities would have been obscured. Here, the idea of rotational symmetry of the impeller is supported by what is *not* observed in the results.

## 4. Conclusions

PC-PSP based on PtTFPP on a titanium oxide base-coat has been used to measure pressure on the rotating blades of a radial compressor with an inlet diameter of 51 mm at rotational speeds up to 50 krpm using the life-time method. Laser light provided by a diode laser was used to excite the paint and the luminescent emission was captured using a photomultiplier tube. The temperature sensitivity of the paint was taken into account by measuring the temperature with an IR camera directly after each run. Even though spatially resolved temperature measurements have not been used to correct the PSP we argue that the errors caused by local temperature variations are small over the tested measurement range.

It has been shown that the proposed scanning-laser method can be used for mapping the surface pressure on a rotating compressor impeller. The measured pressure distribution is found to be in accordance with the pressure increase over the compressor. Time-resolved measurements were able to clearly detect the frequency of the flow at surge condition.

## Figures and Tables

**Table 1:** Tested cases showing rotational speed *ω*, mass flow $\dot{m}$, stagnation pressure ratio $p_{02}/p_{01}$ over the compressor, and median blade temperature $\tilde{T}$.

Case	1	2	3	4	5	6	7	8	9	10
*ω* (krpm)	0	30	30	30	30	10	20	40	50	0
$\dot{m}$ (g· s${}^{-1}$)	0	68	43	21	5.5	26	33	71	93	0
$p_{02}/p_{01}$	1.00	1.06	1.08	1.09	1.08	1.01	1.03	1.13	1.21	1.00
$\tilde{T}$ (${}^{\circ }$C)	29.0	30.4	32.1	35.9	40.1	31.7	31.7	34.9	39.2	44.8
